# Assessing robustness against potential publication bias in Activation Likelihood Estimation (ALE) meta-analyses for fMRI

**DOI:** 10.1371/journal.pone.0208177

**Published:** 2018-11-30

**Authors:** Freya Acar, Ruth Seurinck, Simon B. Eickhoff, Beatrijs Moerkerke

**Affiliations:** 1 Department of Data Analysis, Faculty of Psychology and Educational Sciences, Ghent University, Ghent, Belgium; 2 Institute of Systems Neuroscience, Medical Faculty, Heinrich Heine University Düsseldorf, Düsseldorf, Germany; 3 Institute of Neuroscience and Medicine, Brain & Behaviour (INM-7), Research Centre Jülich, Jülich, Germany; University of Texas at Austin, UNITED STATES

## Abstract

The importance of integrating research findings is incontrovertible and procedures for coordinate-based meta-analysis (CBMA) such as Activation Likelihood Estimation (ALE) have become a popular approach to combine results of fMRI studies when only peaks of activation are reported. As meta-analytical findings help building cumulative knowledge and guide future research, not only the quality of such analyses but also the way conclusions are drawn is extremely important. Like classical meta-analyses, coordinate-based meta-analyses can be subject to different forms of publication bias which may impact results and invalidate findings. The file drawer problem refers to the problem where studies fail to get published because they do not obtain anticipated results (e.g. due to lack of statistical significance). To enable assessing the stability of meta-analytical results and determine their robustness against the potential presence of the file drawer problem, we present an algorithm to determine the number of noise studies that can be added to an existing ALE fMRI meta-analysis before spatial convergence of reported activation peaks over studies in specific regions is no longer statistically significant. While methods to gain insight into the validity and limitations of results exist for other coordinate-based meta-analysis toolboxes, such as Galbraith plots for Multilevel Kernel Density Analysis (MKDA) and funnel plots and egger tests for seed-based d mapping, this procedure is the first to assess robustness against potential publication bias for the ALE algorithm. The method assists in interpreting meta-analytical results with the appropriate caution by looking how stable results remain in the presence of unreported information that may differ systematically from the information that is included. At the same time, the procedure provides further insight into the number of studies that drive the meta-analytical results. We illustrate the procedure through an example and test the effect of several parameters through extensive simulations. Code to generate noise studies is made freely available which enables users to easily use the algorithm when interpreting their results.

## 1. Introduction

Functional magnetic resonance imaging (fMRI) continues to contribute greatly to the knowledge about the location of cognitive functions in the brain [1]. Neuroimaging studies are valuable sources of information for functional organization of the brain but often face substantial challenges. A huge variety in employed experimental conditions and analytical methods exist, possibly leading to inconsistencies across studies and paradigms. Several different implementations and task contrasts might be applied while exploring a certain paradigm, using different analysis toolboxes, pipelines and statistical thresholds. Furthermore, fMRI studies are relatively expensive which often limits the size of studies [2] leading to low statistical power to detect true activation [3]. Further progress in understanding human brain function will therefore also require integration of data across studies using meta-analyses, which can increase power to detect a true effect and allows to assess the replicability or consistency of activated regions across labs and tasks [4–5].

**Fig 1:**
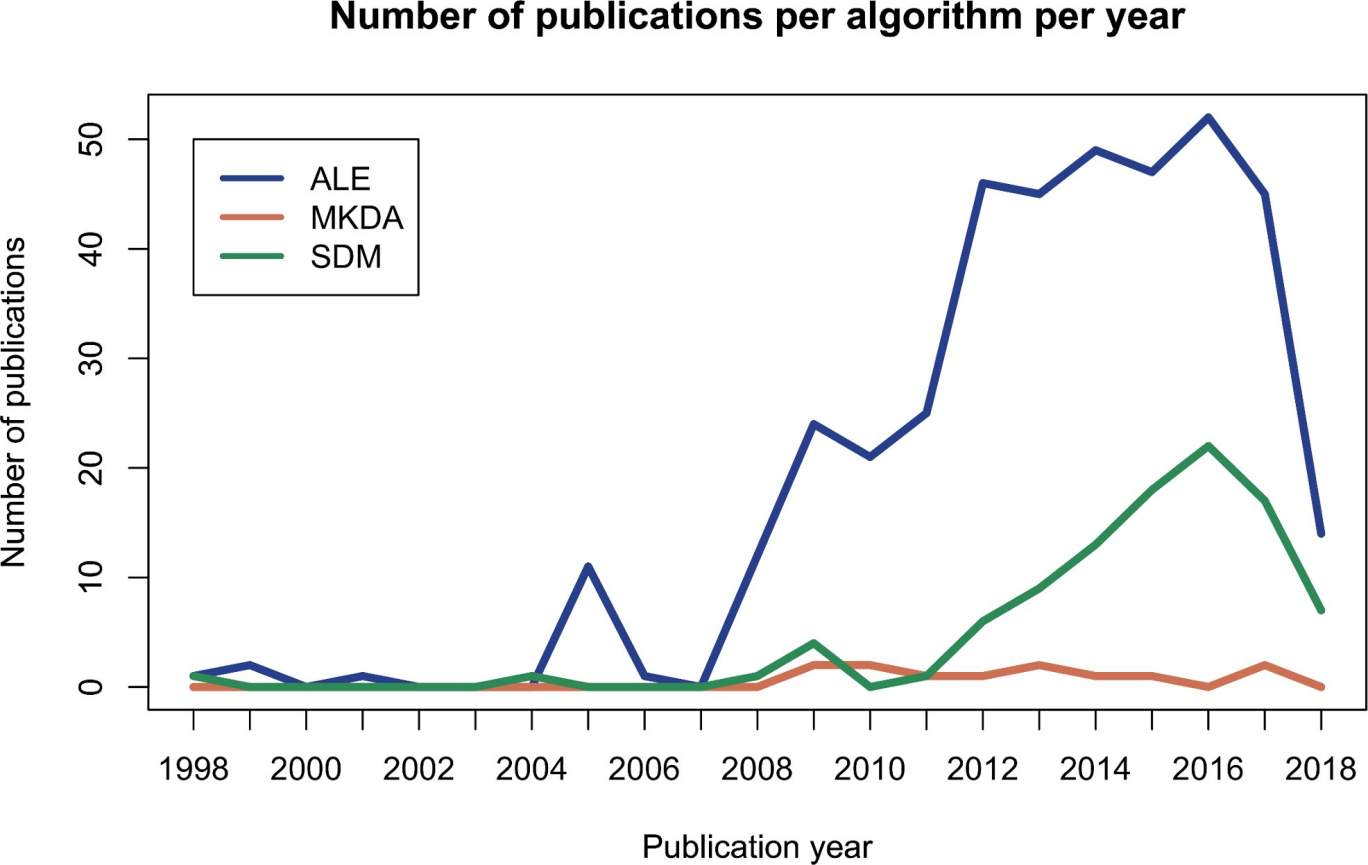
Number of publications per coordinate-based meta-analysis algorithm. Result after a web of science search executed on June 1, 2018. On web of science search terms were ALE meta-analysis for the ALE-algorithm, MKDA meta-analysis for the MKDA algorithm and SDM meta-analysis for the seed-based d mapping algorithm.

fMRI studies capture information about more than 100.000 voxels in the brain, however, the most prevailing trend to report results remains providing only the coordinates of statistically significant local maxima (called peaks or foci) [6]. Several coordinate-based meta-analysis techniques have been developed to combine results of studies that employ this censored reporting of results. First Activation Likelihood Estimation (ALE) [7–9] was developed, which is most frequently used (see Fig 1). Later on, Seed-based d Mapping [10] and multilevel kernel density analysis (MKDA) [5] arose. Other methods include model-based procedures [11–13] and parametric coordinate-based meta-analysis [14].

ALE and MKDA only use the location (xyz-coordinates) of local maxima reported by the individual studies and determine at which location the convergence of foci is larger than can be expected by chance. Seed-based d mapping additionally takes peak height into account and offers the possibility of entering entire t-maps into the meta-analysis. GingerALE [7–9] is a tool that implements the ALE algorithm and is accompanied by the BrainMap database [15–17], which provides an advantage for using ALE. The BrainMap database is a collection of over 3000 papers, containing information about e.g. experimental conditions, subjects and allows to extract peak locations. It is important to point out that peak locations extracted from this database can of course also be used as input for other algorithms, such as MKDA and seed-based d mapping. However, peaks from the BrainMap Database are not optimal for use with seed-based d mapping because peak height is not stored in the BrainMap Database, while seed-based d computes effect sizes based on peak height.

Meta-analyses play an important role in aggregating research findings to form a comprehensive overview and to build cumulative knowledge. Such analyses guide further advancement of theory and research. It is therefore key to rigorously use meta-analytical techniques but researchers should also pay attention to an adequate interpretation of results [18] in the light of possible pitfalls such as publication bias, which occurs when the results of published and unpublished studies differ significantly.

Regardless of the area of research, statistically significant findings are more likely to get published than studies with non-significant results [19] implying that the latter remain largely hidden. This is known as the file drawer problem and is a specific form of publication bias [20–22]. The file-drawer problem can affect the outcome of a meta-analysis by overestimating effects, as no contra-evidence is taken into account. Rosenthal proposed a measure that allows to assess the stability of meta-analytical results [21]. The statistic that is calculated is the “Fail-Safe N” which is the maximum number of studies with an effect of 0 that can be added before meta-analytical conclusions are altered. This was later adapted by Hsu [23] to the maximum number of studies with nonsignificant findings that can be added to a meta-analysis before the conclusions no longer hold. When focusing on the file-drawer problem, it quantifies the number of null studies (i.e. studies without statistically significant results) that are needed to alter a meta-analytical result from statistically significant to statistically non-significant. The Fail-Safe N functions as a measure of robustness as it quantifies the number of studies that are needed to change results, with a low number being indicative for low robustness. In that sense, it can be used as an indication for how robust results are against publication bias. If the expected number or percentage of null studies that remain in the file drawer is known, it is possible to determine whether results remain valid if these null studies would be entered into the meta-analysis. The measure provides an addition to the interpretation of results because it helps in the assessment of the confidence that can be placed in them [18] by showing how stable they remain in the presence of unreported information that may differ systematically from the information that is included.

In neuroimaging literature, it has been shown that there is an excess of positive findings in literature [24, 25]. This stems from different potential sources. David et al. specifically mention selective outcome and analysis reporting bias, manipulation of parameters (such as lenient thresholding) and under-reporting of null results (file drawer) [26]. In this paper, we focus on the latter and more specifically on the robustness of results in the potential presence of file drawer problem. While literature on publication bias in neuroimaging remains limited, evidence for its existence has been found [27].

In the current paper, we focus on the interpretation of meta-analytical results of ALE. We propose a measure for robustness against potential publication bias that is an adaptation of the classical Fail-Safe N. Through an algorithm for computing this measure for ALE meta-analyses, we aim to provide a means to evaluate the stability of results which provides insight into robustness should the file drawer problem be present. The techniques and results are specific to ALE but the basic principles are generalizable to be used with other methods as well. The general idea is to add studies that do not report activation foci in a target area to an existing meta-analysis to determine the amount of contra-evidence that is needed before spatial convergence of foci over studies in that area is no longer statistically significant. The studies with contra-evidence are called “noise studies” as they contain foci that are randomly distributed throughout the brain (as opposed to foci that converge to particular regions). A more detailed explanation can be found in section 2.4. It should be noted that both the classical and adapted Fail-safe N measures are not intended to indicate whether publication bias is present or not. Despite this, we argue that the measure is a useful addition in reporting meta-analytical results while it is conceptually relatively easy to obtain.

The recent mass simulation paper of Eickhoff et al. provides users with clear and motivated guidelines for performing a meta-analysis with the ALE algorithm [7–9, 28]. These guidelines include employing cluster-level family-wise error correction for moderate effects and a set size of at least 20 studies. With the use of an adapted Fail-Safe N measure, we aim to complement these general guidelines by providing an added value to the interpretation of brain regions selected as statistically significant by the ALE algorithm. While a high number indicates results that remain stable even if many noise studies are in the file drawer, a number that is excessively high, exposes another potential problem. It may be the case that some studies in a meta-analysis have an extensive influence on final results. Researchers should therefore also consider the contribution of individual studies in reporting results. Hence, in this setting, the measure provides insight into robustness against publication bias as well as individual study contribution on final results.

In the next sections, we introduce and illustrate the algorithm to calculate the adapted Fail-Safe N for the ALE algorithm with an example meta-analysis and perform extensive simulations to test the influence of number of peaks, sample size and thresholding method on the outcome of the Fail-Safe N. Based on the algorithm outlined in the paper, we also provide a script that allows a researcher to generate noise studies based on the specific properties of their original meta-analysis, namely sample sizes and number of peaks of the individual studies. Researchers can use these noise studies to compute a Fail-Safe N adapted to their specific meta-analysis.

## 2. Material and methods

In this study, we propose a measure for robustness against potential publication bias for coordinate-based (CBMA) meta-analyses. We will focus explicitly on ALE-meta-analyses. In the remainder of the paper, we will refer to the measure of robustness as the Fail-Safe N (FSN) as it is inspired by (but not identical to) this measure that was introduced in classical meta-analysis literature. The original FSN [21] represents the required number of findings with an effect of zero to change the conclusions of the meta-analysis under the assumption that these findings are still in the “file drawer”.

In this section, we first briefly describe some general principles of coordinate-based meta-analyses and indicate which tools for assessing publication bias are already available. Then we briefly describe the ALE procedure in more detail before conceptualizing the FSN for use in an ALE meta-analysis. We refer interested readers to the appendix for a brief discussion of the original FSN for classical meta-analyses. While conceptually similar, its implementation within the context of CBMA meta-analyses, and ALE in particular, differs substantially from this original FSN. Lastly, we present an adaptive algorithm to obtain the FSN to restrict the computations that are needed.

### 2.1 Coordinate-based meta-analysis

Performing a meta-analysis of fMRI studies poses an extra challenge as typically only locations of activated peaks are reported. Different algorithms have been developed to conduct a meta-analysis on peak locations and in this section, we will briefly explain the three most used, seed-based d mapping, the ALE algorithm and MKDA.

Seed-based d mapping is most similar to classic meta-analysis as it computes a weighted average of effect sizes for each voxel. Effect sizes for every study are derived from peak t-statistics or thresholds employed in the individual studies. The ALE algorithm and MKDA are different from seed-based d mapping as they look at the spatial convergence of foci instead of peak height. These algorithms construct kernels of spatial uncertainty around reported peaks and look at the overlap between kernels to determine at which location spatial convergence is larger than can be expected by chance. This rationale differs strongly from classical meta-analyses.

### 2.2 Assessing publication bias in coordinate-based meta-analysis

Publication bias occurs when the results from published and unpublished studies differ systematically. Publication bias becomes problematic for meta-analysis when studies that are included in the meta-analysis are a selective subset of all studies (because, for instance, some studies are not available) and hence are not an accurate representation of executed research. If studies are unavailable because they failed to reach publication this is called the file drawer problem, because these studies remain in the file drawer.

Other robustness and publication bias assessment methods have been developed for CBMA methods other than the ALE algorithm. Seed-based d mapping has the most exhaustive number of possibilities to gain insight into the validity and limitations of results by means of sensitivity analyses and procedures to assess the presence of publication bias. As seed-based d mapping is most similar to a classical meta-analysis it is more straightforward to implement classic assessment methods. One specific form of publication bias is small sample bias, a phenomenon where studies that employ small sample sizes are more likely to report biased results. Assessing small sample bias can be done by constructing a funnel plot [29–30] or performing an Egger test [31–32] (at voxel or region level). A funnel plot is a scatter plot of effect size against a measure of precision (such as sample size or the inverse of the standard error) [30]. These plots enable to assess the presence of small sample bias (in which case smaller samples are associated with higher peaks). Small sample bias is a possible manifestation of publication bias but can also be caused by other issues such as heterogeneity in effect sizes. The Egger regression test estimates funnel plot asymmetry and can therefore be used as an indicator for the presence of publication bias [32]. fMRI studies exhibit great heterogeneity, stemming from the way experiments are constructed, carried out, analyzed and reported [2]. Funnel plots and Egger tests are highly sensitive to heterogeneity, rendering it difficult to interpret their results in the context of a CBMA.

Seed-based d mapping also allows to perform sensitivity analyses using jackknife-analyses [33] or leave-one-out analyses [34] to study how replicable the original findings are. In these procedures, the meta-analysis is repeated with all studies but one to test the stability of the results. If the region of interest remains significant in all or almost all repetitions it can be concluded that the significance of that region is highly replicable. However, one should be aware that failure to replicate the results in one repetition does not automatically implicate high replicability but can indicate that clusters are only due to a small set of studies. If subgroups are present in the dataset, subgroup analyses can be performed to assess whether findings are comparable over subgroups. All of these methods have been implemented in the toolbox for seed-based d mapping (retrieved 30 August 2018 from https://www.sdmproject.com/).This is possible because seed-based d mapping proceeds voxelwise and converts test statistics to effect sizes and subsequently computes a weighted average of effect sizes and therefore resembles classical meta-analyses largely. Applying these methods directly to other CBMA algorithms (such as MKDA and ALE) is unfortunately not possible.

MKDA on the other hand resembles the ALE algorithm as it only takes peak location into account. In MKDA it is possible to construct adapted Galbraith plots. Here, for regions of interest, the presence (yes 1 or no 0) of activation peaks in that region for study each is plotted as a function of sample size. Subsequently a logistic regression is fitted that should pass through the intercept if no small sample bias is present.

### 2.3 ALE meta-analysis

**Fig 2:**
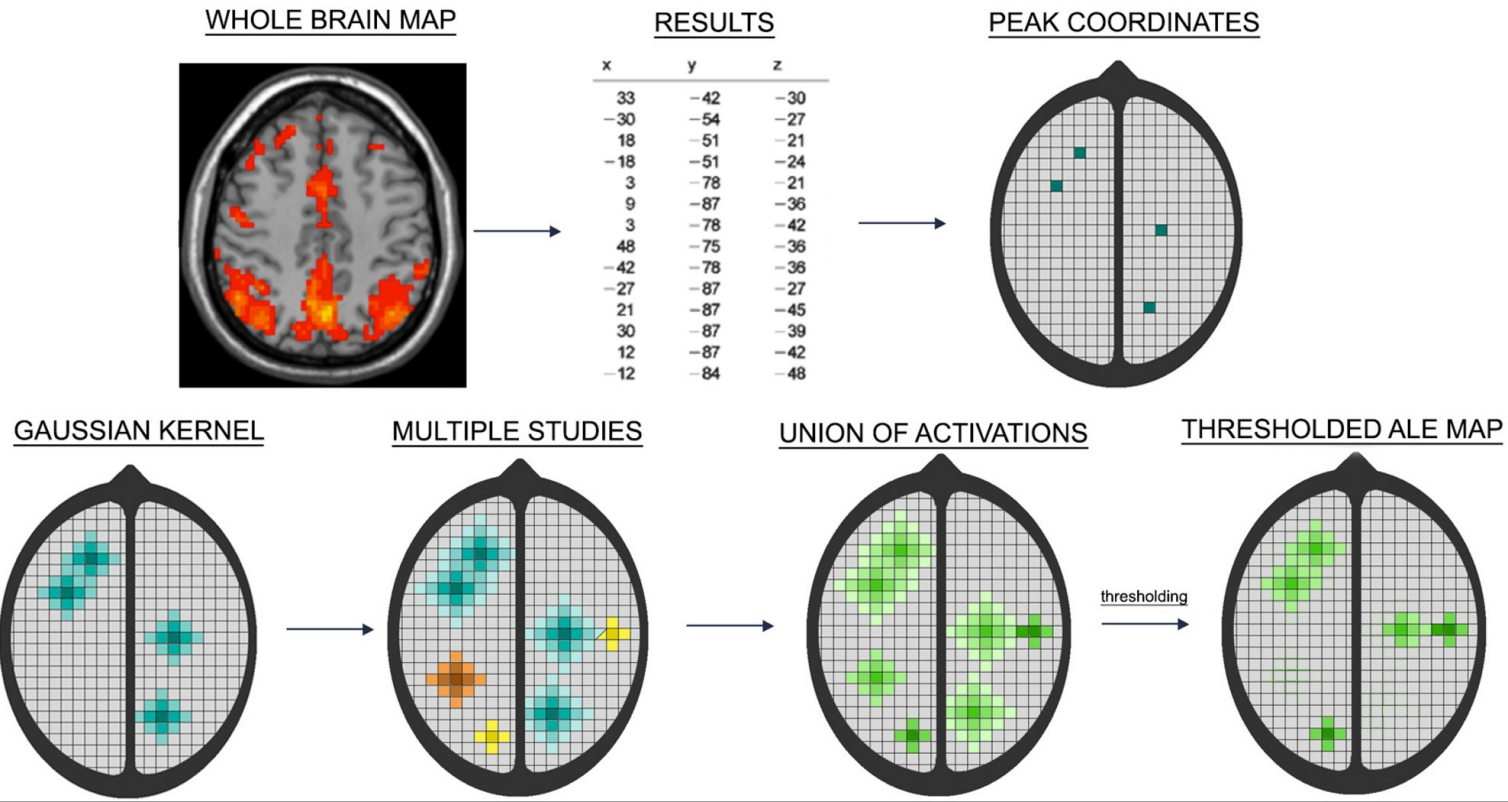
Step-by-step overview of the ALE algorithm. From an entire Statistical Parametric Map (SPM) usually only peak coordinates (foci) are reported. These are entered in an empty brain for every study individually. Spatial uncertainty is accounted for by constructing Gaussian kernels around the foci of which the size depends on the sample size of that study (smaller studies have more spatial uncertainty and larger kernels). The result of this process is a set of MA-maps. An ALE-map is constructed by calculating the union of the MA-maps. Eventually the ALE-map is thresholded to determine at which locations the convergence of foci is larger than can be expected by chance.

The ALE procedure consists of determining whether the spatial convergence of foci or local maxima across studies is larger than can be expected by chance. An overview is given in Fig 2. While the result of an fMRI data analysis is a statistical parametric map, often only peak locations are reported. In the ALE algorithm, these reported foci are entered into an empty brain by assigning them a value of 1, all other voxels have a value of 0. For every study a map is constructed with its reported foci. In a next step this value of 1 is smeared out by smoothing to neighbouring voxels by means of a Gaussian kernel of which the size depends on the sample size of the study. Small studies have less statistical power and more spatial uncertainty and therefore kernel size increases with decreasing sample size. The resulting map is called a modelled activation (MA) map and represents for each voxel the probability of one of the activation foci being present in that voxel. Subsequently the MA-maps are combined into one ALE-map by taking the union of probabilities (Eq 1). For every voxel with coordinates (x, y, z), the ALE value in the map is the result of taking the union of the MA-values of that voxel over the k studies entered into the meta-analysis.

$$ALE_{xyz}=1-\prod_{i=1}^{k}\left(1-MA_{kxyz}\right)$$ (1)

Thresholding of the resulting ALE map can be done both voxelwise (uncorrected, or with multiple testing corrections to control the False Discovery Rate FDR or the Family Wise Error FWE rate) or on cluster-level. In the case of voxel-level thresholding, a null distribution is constructed based on the null hypothesis that foci are randomly spread throughout the brain. This null distribution contains all possible ALE values that can be obtained by making all possible combinations of voxels in the MA-maps inside the mask. We can illustrate this with a simplified example. Imagine a meta-analysis of 3 studies, each with 11 voxels inside the mask. The first value in the null distribution is obtained by computing the ALE-value resulting from the combination of the MA-values in voxel 1 of study 1, voxel 1 of study 2 and voxel 1 of study 3. The second value is the result of the combination of voxel 1 of study 1, voxel 1 of study 2 and voxel 2 of study 3. This process is repeated until all 1331 possible ALE-values are known. The resulting vector of possible ALE-values can be visualised as a histogram and is used as a null distribution to obtain voxel-level thresholds. On top of a voxel-level threshold, a cluster-level threshold can be added by randomly distributing the MA-values throughout the brain, performing the same voxel-level threshold and listing the size of the largest resulting cluster. This process is repeated (e.g. 1000 times) and the distribution of cluster sizes is used to compute a cluster-level threshold. The authors of the ALE algorithm advise to employ a voxel- or cluster-level family-wise error correction (FWE) [28].

### 2.4 The FSN for an ALE meta-analysis

In this paper, the FSN is defined as the amount of contra-evidence that can be added to a meta-analysis before the results of that meta-analysis are changed. The FSN can be obtained for each region that survives thresholding in an ALE meta-analysis. Note that it is not possible to enter studies without any peaks in the ALE algorithm because adding studies without foci does not change the results of the meta-analysis. In Eq (1), it can be seen that if an MA_kxyz_ of 0 is entered into this formula, the second part is multiplied by 1 and hence the ALE-value is not affected. In addition, the null distribution of the ALE-values also remains unaltered. This implies that ALE is insensitive to null studies. We are however interested in assessing the number of studies that do not report foci in a region of interest that can be added before spatial convergence of foci in the region is no longer statistically significant but such studies can report foci in other regions. We therefore conceptualize contra-evidence for studies in the ALE meta-analysis as noise studies that report foci randomly distributed throughout the brain. ALE meta-analysis findings change when such noise studies are added into the meta-analysis. Eickhoff and colleagues have shown that adding random coordinates to an ALE meta-analysis has a limited impact on the resulting ALE values, even if they overlap with the cluster of interest [28]. The threshold however does change. By adding foci, the null distribution used to compute the threshold changes, with only larger ALE values surviving statistical thresholding.

Currently, the FSN measure is rarely employed in classical meta-analyses. The measure has been criticized for a variety of reasons [20, 35–36] which essentially boil down to having different possibilities for conceptualizing a null finding and its focus on statistical instead of practical significance. These critiques do not apply directly to the context of ALE coordinate-based meta-analyses. First, the definition of null findings can be defined rather unambiguous as ALE only requires the location of peak voxels as input. Randomly sampled peaks show no convergence of foci and are therefore an adequate representation of a study with null findings with respect to a specific region. Secondly, the input on which ALE meta-analyses are based is in essence binary (active versus non-active) due to censored reporting based on statistical significance. This precludes the calculation of effect sizes that are employed in classical meta-analysis.

The FSN is a measure of robustness against potential publication bias. It indicates the stability of meta-analytical results when results that may differ systematically from the other studies are included in the meta-analysis. If studies are added that remain otherwise hidden in the file drawer, researchers can assess how the file drawer problem may affect their results. A higher FSN indicates more stable results and hence a higher robustness. To aid interpretation of results, a minimum FSN that indicates vulnerability to the file-drawer effect is needed. Rosenthal provided a guideline of 5k + 10 (with k the number of studies in the original meta-analysis) as minimum for the FSN, but this is specifically adapted to behavioural studies [21]. It can be expected that the number of studies remaining in the file drawer is lower for fMRI studies than for behavioural studies as fMRI studies are often more costly and time-demanding. It is key that a researcher determines a minimum FSN specific for the area of research. A recent paper estimates the number of studies or experiments with no foci that remain unnoticed in fMRI meta-analyses [37]. Using the Brainmap database with data on the number of foci reported for specific contrasts or experimental conditions that are tested in fMRI studies, the authors predict for different areas of research the number of studies that report no local maxima. The results indicate that the number of missing contrasts is at least 6 for every 100 studies published, but the number can be as high as 50 out of 100 studies for some research domains. In addition, the authors provide confidence intervals for the estimators for the missing contrasts. While the estimates [37] may be not all-encompassing as assessing the amount of hidden studies remains a great challenge, these results can aid in determining a minimum FSN but should not be the sole driving force for this.

It has further been shown that some studies may have a large impact on final results [28]. Through massive simulations the authors showed that, even if appropriate thresholding was applied, the two most dominant experiments, i.e. the experiments that contributed most to the observed ALE-value in the cluster of interest, in a meta-analysis account for more than 60% of the total ALE score of a significant cluster. More specifically, if e.g. 25 studies are entered into a meta-analysis, the two most dominant experiments, which is less than 10% of the experiments entered, contribute to between 65% and 75% of the total ALE score of the cluster. For each significant cluster selected by the ALE algorithm, it is important to look at the contribution of each individual study to that cluster. If the FSN for a given region is very high, this means that few studies drive the results for that cluster. For example, when 3 studies out of 10 studies contribute to the results of a specific cluster, there is a contribution from 30% of the studies. However, when the FSN for that same analysis is larger than 100, this means that the cluster remains significant even if less than 3% of the studies contribute to these results. This is indicative for highly influential studies. To detect such cases using the FSN, a practically relevant upper boundary can be defined by delineating the minimum percentage of studies that contribute to a given effect. For example, if 20 out of 50 studies in a meta-analysis contribute to a cluster and one requires that at least 5% of the studies contribute to the cluster of interest, the upper boundary of the FSN is 350.

### 2.5 Algorithm to compute FSN for an ALE meta-analysis

To compute the FSN, a researcher first needs to generate a set of noise studies for which the results can be added to an existing meta-analysis. We provide R code that can be used to generate noise studies. This code is freely available on GitHub (https://github.com/NeuroStat/GenerateNull). Once a set of noise studies is obtained, these can be added to the list of original studies in the meta-analysis and the meta-analysis can be rerun using this expanded list of studies. The same toolbox (e.g. GingerALE) as for the original analysis can be used.

To generate null studies, users first need to read in the same list of foci that was entered in ALE. From this list, the number of peaks and participants per study are saved, each in an individual vector. The researcher has the possibility to indicate the number of noise studies he or she wants to generate. If no number is entered 10k (with k the number of experiments in the original meta-analysis) noise studies are generated. The number of peaks and number of participants of these noise studies are randomly sampled from the previously saved vectors. If the original meta-analysis only contains studies with less than 20 subjects, the noise studies will also have less than 20 subjects. Likewise, if the studies in the meta-analysis reported between 10 and 20 foci, the simulated noise studies will also report between 10 and 20 foci. A lot of variability in sample size and number of reported peaks exists. By reusing the sample sizes and number of peaks we assume that studies that remain in the file drawer are similar to the published studies with respect to these parameters. If there is however evidence that the studies remaining in the file drawer have specific sample sizes or number of peaks, it is possible to change these parameters in the R program. To ensure that all peaks lie within the grey matter of the brain, the location of these peaks are sampled from within the mask used by the ALE algorithm to anatomically limit meta-analyses to grey matter regions.

Determining the FSN is an iterative process as each time, a different number of noise studies are added to the meta-analysis. Performing multiple ALE meta-analyses is a time-demanding process. Pre-specifying boundaries between which the FSN will be sought reduces the number of steps dramatically. The lower boundary can be set to the predefined minimum FSN while the upper boundary can be determined by pre-defining the minimum percentage of studies that contribute to a given effect.

In many cases, it may be sufficient to know whether the FSN is lower than the lower bound (indicating non-robustness against publication bias) or whether the FSN is higher than the upper bound (indicating that results are driven by a small number of influential studies). When the FSN lies between the boundaries, it means that results are sufficiently robust and are supported by at least the desired minimum of contributing studies. To know where the FSN is situated in comparison to the boundaries, only two additional meta-analyses need to be run: a meta-analysis for which the number of noise studies equals the lower boundary and a meta-analysis where the number of noise studies equals the upper boundary.

**Fig 3:**
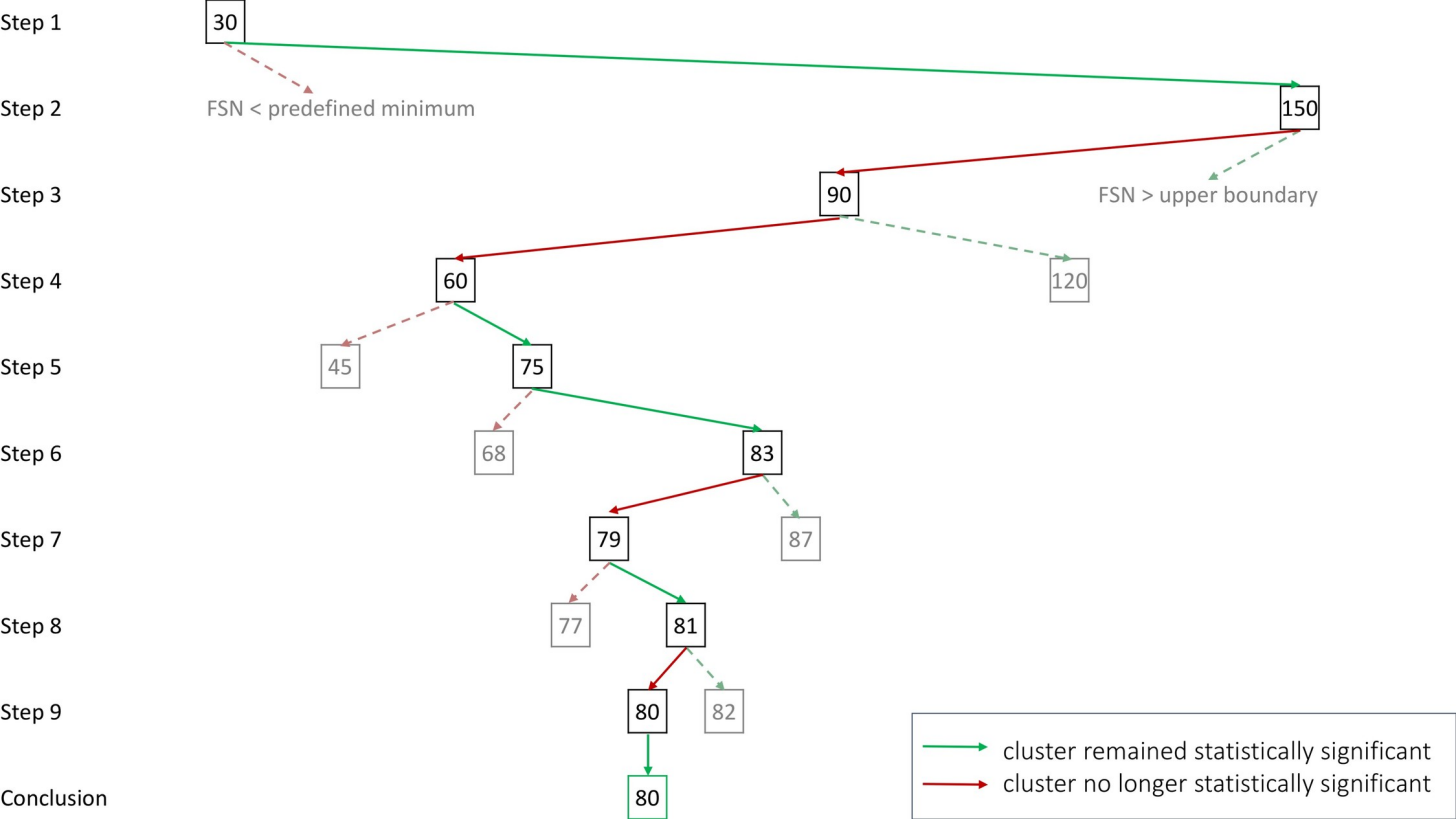
Illustrated example of the algorithm used to compute the FSN of a meta-analysis of 15 experiments (k = 15). The predefined lower boundary is set to 30 (2k) and the predefined upper boundary is set to 150 (10k). First an ALE meta-analysis is run while a number of noise studies equal to the lower boundary is added to the original dataset. If the cluster is no longer statistically significant the FSN is smaller than the predefined minimum. If the cluster is still statistically significant after these noise studies are added, a second analysis is run by adding the number of noise studies defined by the upper boundary. If the cluster is still statistically significant the FSN is larger than the upper boundary, if it is no longer statistically significant the FSN lies somewhere between the predefined lower boundary and upper boundary. To reduce the number of steps needed to be taken to determine the FSN the number of noise studies added in the next step is the average of the lower boundary and upper boundary FSN. Is the cluster still statistically significant, then the lower boundary is changed to the number of noise studies that was added. If the cluster is no longer statistically significant this number now becomes the upper boundary. This process is repeated until the lower boundary and upper boundary are only one number apart, and the FSN is known. In this example the FSN is equal to 80.

Computing the exact FSN is however more computationally intensive. To reduce the number of computational steps we present a step-wise algorithm that is usually able to compute the FSN in 15 steps or less. An illustrated example of the algorithm can be found in Fig 3. Here we provide pseudocode to illustrate the algorithm:

1. Run an ALE meta-analysis of interest containing k studies (in the example k = 15) and choose a specific statistically significant cluster for which the FSN will be determined.
2. Re-run the meta-analysis with the original studies with m noise studies added where m is the pre-specified lower boundary for the FSN (in the example m = 30).
  Possible results:
  - The cluster is no longer statistically significant: STOP. Adding m studies alters the significance of your results. This indicates that results may not be robust when bias due to missing (noise) studies in the meta-analysis is present.
  - The cluster remains statistically significant: proceed to step 3.
3. Add M noise studies with M the pre-specified upper boundary for the FSN (in the example M = 150).
  Possible results:
  - The cluster is no longer statistically significant. The FSN lies between the pre-specified lower boundary m and upper boundary M. Set M* = M and m* = m and calculate N as the average of M* and m*. Proceed to step 4.
  - The cluster remains statistically significant: the FSN is higher than the pre-specified upper boundary M. This indicates that results for the cluster may be driven by a small number of studies. STOP or choose a higher upper boundary for the FSN. Set the lower boundary to your currently specified upper boundary. Start again from step 2.
4. Perform a meta-analysis with k activated studies and an addition of N noise studies.
  Possible results:
  - The cluster is no longer statistically significant: remove N- N_0_ noise studies with N_0_ the average of N and m*.
    This replaces N by N_0_, M* by N and m* remains unaltered.
  - The cluster remains statistically significant: add N_1_–N noise studies studies with N_1_ the average of N and M*.
    This replaces N by N_1_, m* by N and M* remains unaltered.
5. Repeat step 4 and continue to add and/or remove noise studies until the FSN is determined.

## 3. Example

### 3.1 Method

To demonstrate the principle of the FSN we replicate a previously executed meta-analysis. We select a meta-analysis on finger tapping [7,38] from the BrainMap database that was used to demonstrate the working of GingerALE [15–17]. In total 38 papers (347 subjects) with a total of 654 activation foci are obtained. The replication differs from the original meta-analysis in one aspect. In the original meta-analysis one study could report multiple experiments with the same subjects, and these experiments would be interpreted as different studies. 73 experiments, coming from 38 studies, were reported. Using correlated data by reporting multiple experiments on the same set of subjects is problematic and induces bias [39,40]. Therefore, in this example all contrasts that stem from the same study with the same set of subjects are reported as one experiment [8]. All data, analysis procedures and results can be found on GitHub (https://github.com/NeuroStat/FailSafeN).

As an example, we base the minimum FSN on results in [37]. It is estimated for normal mapping that a 95% confidence interval for the number of studies that report no local maxima varies from 5 to 30 per 100 published studies. Using the upper bound and the fact that the meta-analysis consists of 38 experiments, a possible estimate for the number of experiments that remain in the file drawer is 11. Therefore, the minimum FSN is defined as 11. Since the number of contributing studies differs for every cluster the upper boundary varies between clusters. For clusters 1 to 10 the number of contributing studies is 33, 29, 19, 19, 14, 14, 11, 9, 10 and 6 respectively. If we aim to have at least 10% study contribution, the upper boundary for clusters 1–7 respectively is equal to 292, 252, 152, 152, 102, 102, 76, 76, 76 and 76.

Foci from the individual studies are obtained from the BrainMap database. The aforementioned R program (https://github.com/NeuroStat/GenerateNull) is used to generate noise studies. After reading in the list of foci the distributions of the sample sizes and number of reported peaks are saved. Based on these distributions noise studies are constructed and saved into a text-file with the correct lay-out for the ALE algorithm. New text-files are created by pasting the desired number of noise studies after the studies of the original meta-analysis. The resulting text-files with lists of foci are entered into GingerALE 2.3.6 and meta-analyses are performed until the FSN is known for every cluster. The analysis is thresholded with a cluster-level FWE correction of p < 0.05 (cluster-forming threshold of p < 0.001, uncorrected), 1000 permutations are performed.

### 3.2 Results

**Fig 4:**
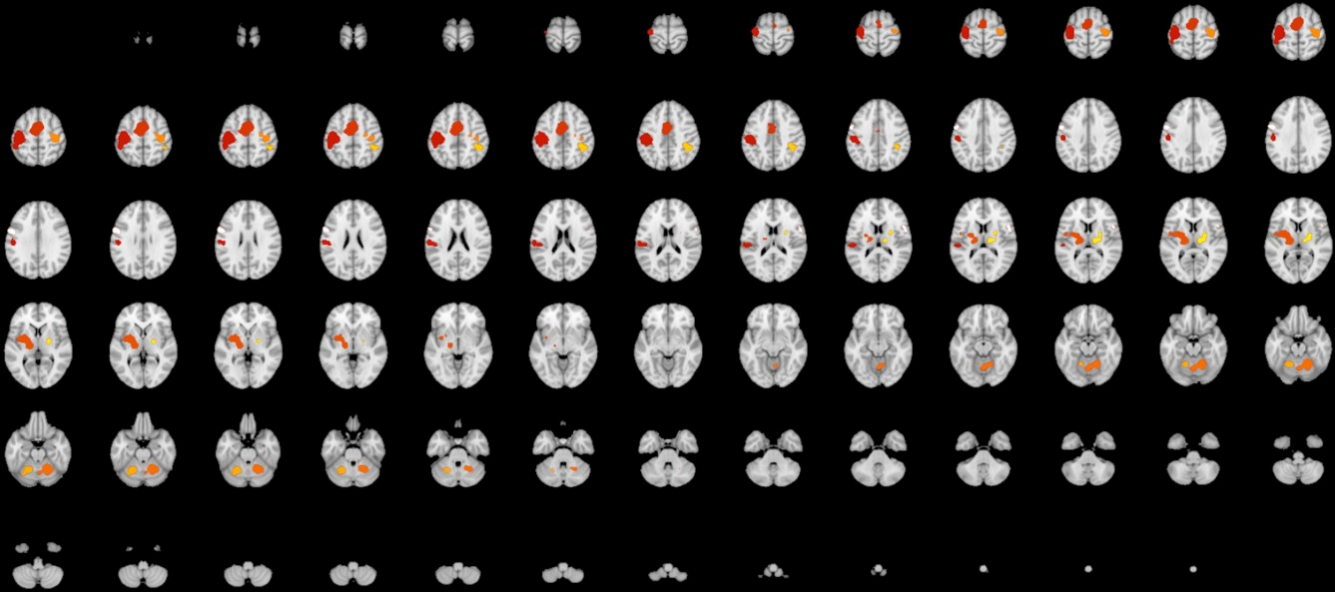
Cluster-level thresholded ALE map of a meta-analysis on finger tapping. ALE maps were computed using GingerALE 2.3.6 with a cluster-level forming threshold of p < 0.05 (cluster-forming threshold p < 0.001, uncorrected), visualised with Mango.

After thresholding 10 statistically significant clusters are found, these are reported in Table 1. The location of these clusters is plotted in Fig 4. The clusters varied in size from 1144mm^3^ to 20488mm^3^. Convergence of foci is found bilaterally in areas responsible for motor functioning, namely the putamen, the primary motor cortex, the premotor cortex and the insular cortex, somatosensory areas, namely the primary somatosensory cortex and areas involved in reading and semantic processing, namely the supramarginal gyrus. In the left hemisphere convergence of foci is detected in the ventral posterior lateral nucleus, while in the right hemisphere convergence of foci was found in the pars opercularis, dentate, globus pallidus and medial dorsal nucleus. These regions are suggested to be involved in the motor aspect of speech and voluntary movement.

**Table 1:** Volume, weighted center and local maxima of the 7 clusters resulting from the ALE meta-analysis.

Cluster number	Volume (mm^3^)	Weighted Center (x,y,z)			Extrema Value	x	y	z	Label
1	20488	-42.4	-23.4	50	0.074209824	-38	-24	54	Left Cerebrum.Parietal Lobe.Postcentral Gyrus.Gray Matter.Brodmann area 3
					0.029201938	-48	-38	52	Left Cerebrum.Parietal Lobe.Inferior Parietal Lobule.Gray Matter.Brodmann area 40
					0.021789065	-52	-26	14	Left Cerebrum.Temporal Lobe.Transverse Temporal Gyrus.Gray Matter.Brodmann area 41
					0.020919967	-52	-22	32	Left Cerebrum.Parietal Lobe.Postcentral Gyrus.Gray Matter.Brodmann area 2
					0.020768775	-60	-22	20	Left Cerebrum.Parietal Lobe.Postcentral Gyrus.Gray Matter.Brodmann area 40
2	12688	-2.5	-2.6	53	0.065038785	-4	-6	52	Left Cerebrum.Frontal Lobe.Medial Frontal Gyrus.Gray Matter.Brodmann area 6
					0.054191217	2	0	52	Left Cerebrum.Frontal Lobe.Medial Frontal Gyrus.Gray Matter.Brodmann area 6
3	8624	-24.6	-8.7	4.7	0.03301656	-32	-4	4	Left Cerebrum.Sub-lobar.Lentiform Nucleus.Gray Matter.Putamen
					0.031805474	-24	-6	4	Left Cerebrum.Sub-lobar.Lentiform Nucleus.Gray Matter.Putamen
					0.03090092	-16	-18	8	Left Cerebrum.Sub-lobar.Thalamus.Gray Matter.Ventral Posterior Lateral Nucleus
					0.016557096	-48	-4	8	Left Cerebrum.Frontal Lobe.Precentral Gyrus.Gray Matter.Brodmann area 6
4	7680	16	-56.5	-21.2	0.047102448	18	-54	-22	Right Cerebellum.Anterior Lobe.*.Gray Matter.Dentate
					0.026701462	2	-62	-16	Right Cerebellum.Anterior Lobe.Culmen.Gray Matter.*
5	4952	35.4	-20.4	56.8	0.027584236	38	-22	58	Right Cerebrum.Frontal Lobe.Precentral Gyrus.Gray Matter.Brodmann area 4
					0.026873175	38	-22	54	Right Cerebrum.Frontal Lobe.Precentral Gyrus.Gray Matter.Brodmann area 4
					0.018016322	26	-16	50	Right Cerebrum.Frontal Lobe.Precentral Gyrus.Gray Matter.Brodmann area 4
6	3504	-21.4	-57.2	-23.8	0.035299618	-22	-56	-26	Left Cerebellum.Anterior Lobe.*.Gray Matter.*
					0.013809228	-34	-56	-30	Left Cerebellum.Anterior Lobe.*.Gray Matter.*
7	3072	41.5	-40.5	45.9	0.031791035	38	-38	44	Right Cerebrum.Parietal Lobe.Inferior Parietal Lobule.Gray Matter.Brodmann area 40
8	2800	20.2	-10.7	8.3	0.025899366	14	-16	10	Right Cerebrum.Sub-lobar.Thalamus.Gray Matter.Medial Dorsal Nucleus
					0.020860475	22	-8	4	Right Cerebrum.Sub-lobar.Lentiform Nucleus.Gray Matter.Lateral Globus Pallidus
					0.016862255	24	-14	8	Right Cerebrum.Sub-lobar.Thalamus.Gray Matter.*
9	2592	-55.5	3.1	30.2	0.025755841	-58	6	26	Left Cerebrum.Frontal Lobe.Precentral Gyrus.Gray Matter.Brodmann area 6
					0.019526139	-56	0	36	Left Cerebrum.Frontal Lobe.Precentral Gyrus.Gray Matter.Brodmann area 6
10	1144	54.4	9.2	12.8	0.017568473	54	12	12	Right Cerebrum.Frontal Lobe.Precentral Gyrus.Gray Matter.Brodmann area 44

* indicates that the area in which the extrema value lie cannot be specified more.

The FSN for every cluster is displayed in Table 2. The FSN exceeds the upper boundary for 6 out of 10 clusters (clusters 1, 2, 3, 4, 5 and 6), indicating a robust convergence of foci in these regions but also indicating that proportionally little studies are needed to obtain this effect. For 3 clusters (clusters 7, 8 and 9) the FSN lies between the lower and upper boundary but for 1 cluster (cluster 10) the FSN is smaller than the predefined minimum. The region covered by this cluster is the pars opercularis. This area is suggested to be involved in semantic tasks and the motor aspect of speech. We see that in the areas like the primary motor cortex, the premotor cortex and the primary somatosensory cortex the FSN often exceeds the upper boundary, while clusters that involve deeper structures such as the putamen have a lower FSN.

**Table 2:** Properties and FSN of the clusters resulting from the meta-analysis.

Cluster number	Volume (mm^3)^	Weighted Center (x,y,z)			Extrema Value	Number of contri-buting studies	FSN
1	20488	-42.4	-23.4	50	0.0742	33	> 292
2	12688	-2.5	-2.6	53	0.0650	29	> 252
3	8624	-24.6	-8.7	4.7	0.0330	19	> 152
4	7680	16	-56.5	-21.2	0.0471	19	> 152
5	4952	35.4	-20.4	56.8	0.0276	14	> 102
6	3504	-21.4	-57.2	-23.8	0.0353	14	> 102
7	3072	41.5	-40.5	45.9	0.0318	11	51
8	2800	20.2	-10.7	8.3	0.0259	9	15
9	2592	-55.5	3.1	30.2	0.0258	10	27
10	1144	54.4	9.2	12.8	0.0176	6	< 11

For every cluster the volume, weighted center, peak ALE-value, number of contributing studies and Fail-Safe N.

## 4. Simulations

### 4.1 Method

**Fig 5:**
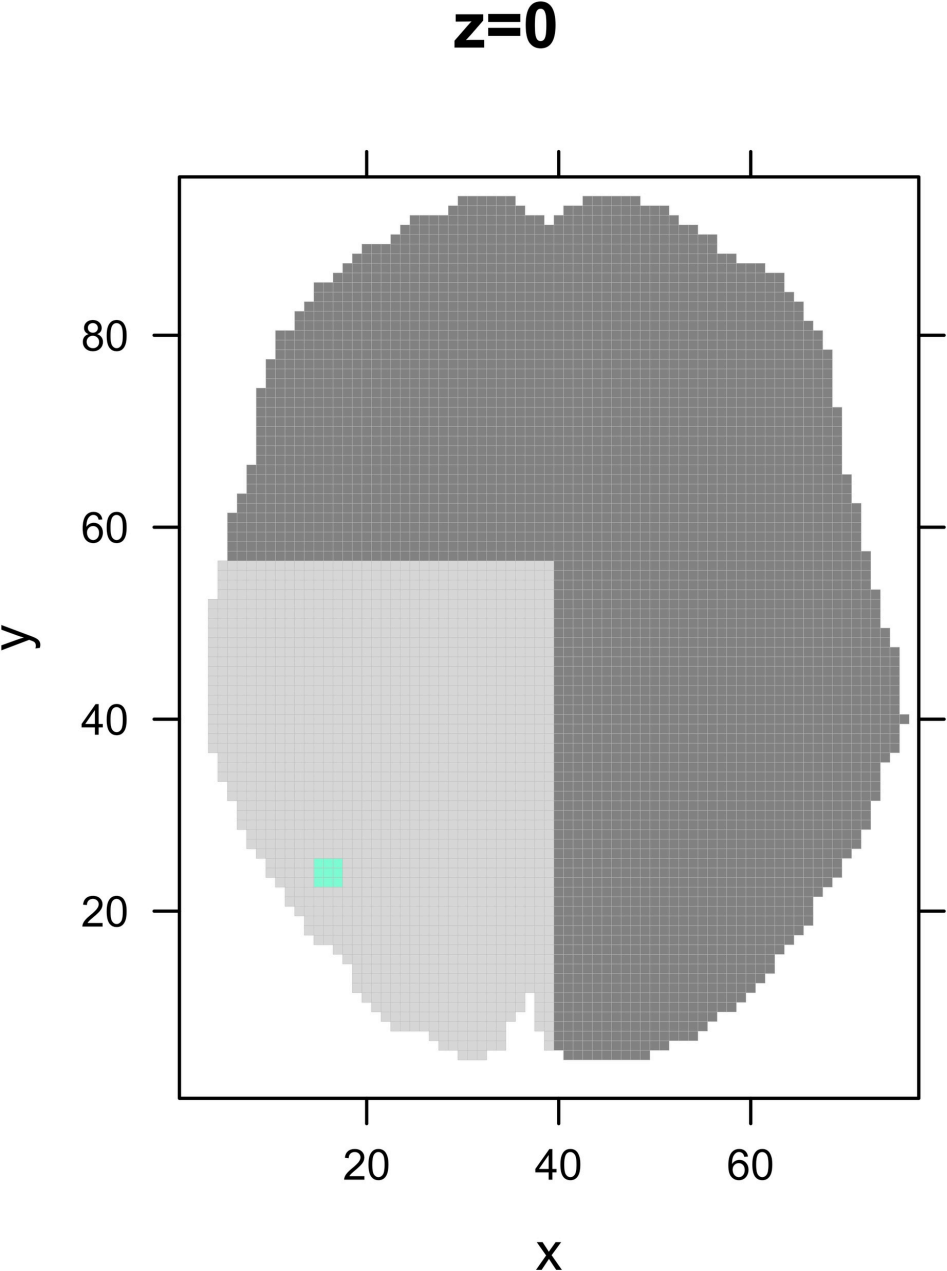
Visualisation of the 4 quadrants the brain is divided into for simulation. The blue dot represents the true location of activation and the peaks of the 3 activated studies are sampled around this location. To avoid interference between the activated and the noise studies, the peaks of the noise studies are sampled from quadrants 2, 3 and 4 (darker shades of grey).

To study the influence of study parameters, such as number of peaks per study, sample size and thresholding method employed by the ALE algorithm, on the result of the FSN, we simulate meta-analysis sets. These sets consist of lists of peaks that are divided into studies. Each meta-analysis set consists of 3 studies that report local maxima close to the location of activation peak, we call these activated studies, and of 100 noise studies that report local maxima elsewhere in the brain. The number of activated studies is arbitrarily chosen and might seem small. However, Eickhoff et al. have shown that as little as 2 experiments often cause more than half of the total ALE value of a cluster [28]. To determine the location of the local maxima, the brain is divided into 4 quadrants (Fig 5). In quadrant 1 the peaks of the 3 activated studies are sampled at a random distance from the location of true activation (x = 46, y = -66, z = -6 in MNI space which is the standard brain space used by the ALE algorithm to map locations in the brain). The distance between the location of true activation and the simulated activation is measured in voxels and sampled from a normal distribution (Distance ~ N(0,1)). This location is randomly chosen. The peaks of the noise studies are randomly selected from a list of voxels inside quadrants 2, 3 and 4 (Fig 5). In that way, false peaks are hypothesised to be randomly distributed throughout the brain while true peaks are hypothesised to lie close to the location of true activation, with a higher chance of being close to the location of true activation than lying further away from it. By working with quadrants, we further ensure that no overlap would exist between true activation peaks and noise peaks, but Eickhoff et al. have shown that influence of overlapping would have been minimal [28].

To assess the effect of number of reported peaks per study 3 scenarios are simulated. In a first scenario, every study reports 1 peak. Although a large number of experiments in the BrainMap database merely report 1 peak, it is unlikely that all studies in a meta-analysis report 1 local maximum. Therefore, we extract the distribution of local maxima that are reported by the studies present in the BrainMap database. The number of reported peaks lies between 1 and 98, with an average of 8, and the median equal to 5. In a second scenario every study reports 8 peaks while in a third scenario the number of peaks per study reported varies and is sampled from the distribution of reported peaks listed by studies included in the BrainMap database.

To evaluate the effect of sample size, 3 copies of the 3000 meta-analysis sets are created. The only difference between these copies is the average sample size. The sample size of the 3 activated studies varies and is drawn from a normal distribution with standard deviation 1 and average equal to 10 (small sample size), 20 (average sample size) or 30 (large sample size). The sample size of the smallest of the 3 activated studies is used as the sample size of the noise studies and remains the same over all 100 noise studies. Small studies use a larger kernel size in the smoothing step and therefore have a larger influence on the end result. By using the smallest sample size for the noise studies, we ensure that the noise studies have at least as much weight on the final result as the activated studies. To compute the FSN, ALE meta-analyses are performed with a varying number of noise studies until the tipping point of statistical significance is found. Data obtained through simulations is used for validation and the Matlab implementation of ALE is used (Eickhoff, personal communication).

We apply 2 different methods for thresholding, namely voxelwise Family-Wise Error Rate (vFWE, p < 0.05) and a cluster-level FWE thresholding (p < 0.05) with uncorrected cluster-forming threshold (p < 0.001). For the voxel-level thresholding methods no additional minimum cluster size is employed. 100 permutations are performed to determine the cluster-level threshold.

**Fig 6:**
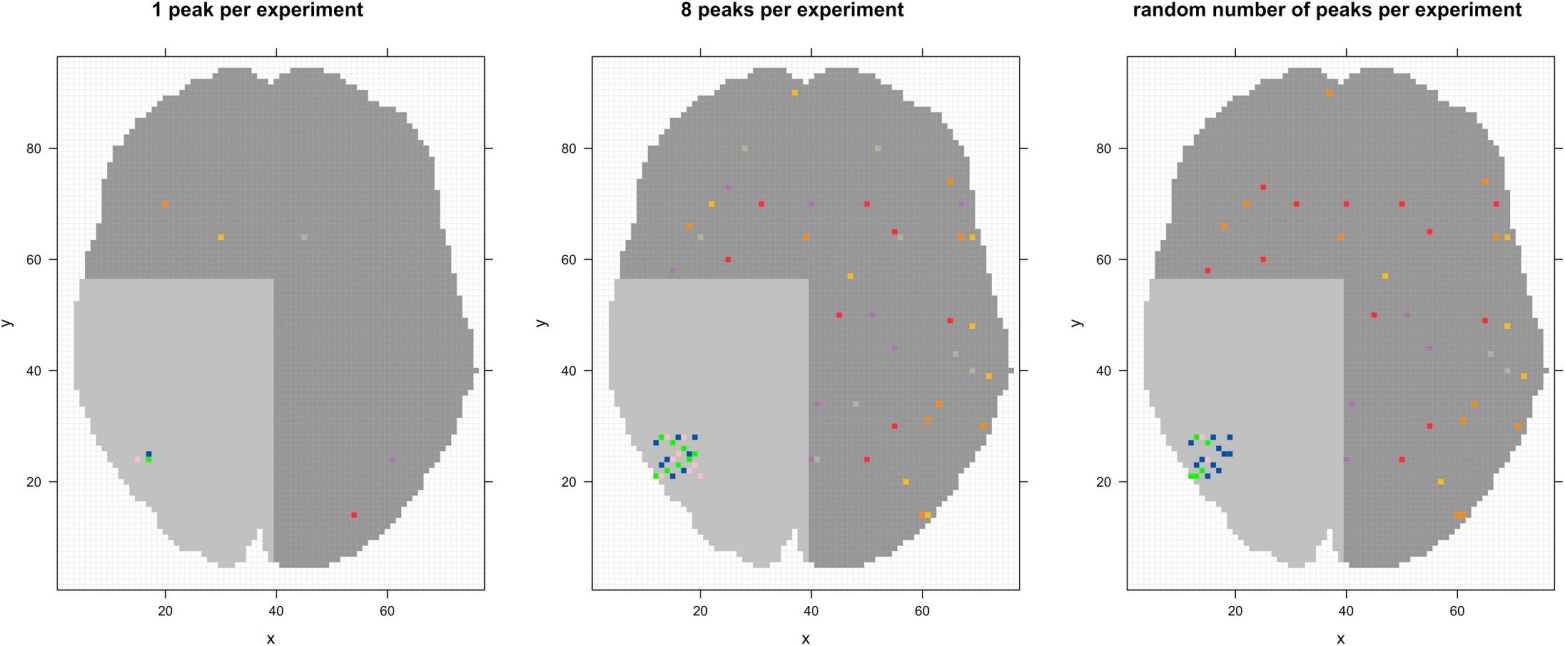
Visualisation of simulation conditions with 3 activated studies and 5 noise studies. The visualisation is simplified to 2D slices with z = 0. In the activated simulations coordinates are entered into 3D space, resulting in a larger spread. In the first pane we see the scenario with 1 peak per study. The peaks from the 3 activated studies lie in quadrant 1, the peaks from the noise studies lie in the other 3 quadrants. The pane in the middle represents the scenario with 8 peaks per study. The activated studies each have eight peaks in quadrant 1, resulting in 24 peaks close to each other. The 5 noise studies each have 8 peaks in the remaining 3 quadrants. Their peaks will never lie in quadrant 1. Due to the random location of peaks some overlap and spurious activation might occur. The influence of this spurious activation is minimal after 1000 simulations. In the right pane the scenario with a random number of peaks is shown. The 3 activated studies have 5, 11 and 2 peaks respectively. The 5 noise studies have peaks in the remaining quadrants, with 2, 12, 4, 11 and 5 peaks respectively.

The simulations are illustrated in Fig 6. In total 18 different scenarios are simulated, for every scenario 1000 simulations are performed. For every one of the 18.000 meta-analyses the FSN is determined by repeatedly adding and removing noise studies until the tipping point of for the cluster of interest is found. Due to computational complexity, we stopped when adding 100 noise studies did not alter results. Datasets and code are available on GitHub (https://github.com/NeuroStat/FailSafeN).

### 4.2 Results

**Fig 7:**
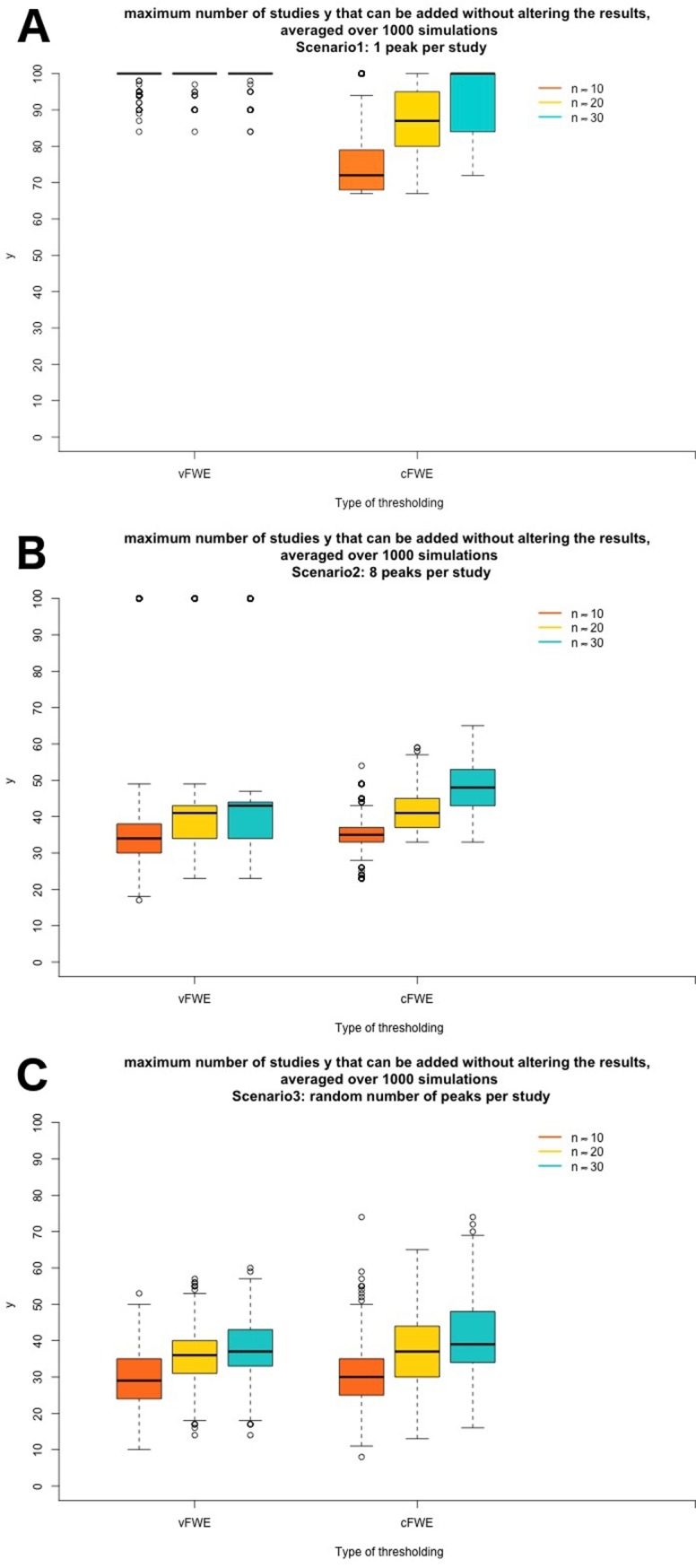
Boxplots of simulations with the FSN in all three scenarios. On the x-axis the thresholding method and average number of participants per experiment can be found. On the y-axis a boxplot of the number of noise studies that can be added to a meta-analysis of 3 studies with activation before the target area is no longer statistically significant is plotted. In panel A results for the scenario with 1 peak per study are shown, in panel B results for the second scenario, with 8 peaks per study and finally in panel C the results for studies with a random number of peaks are shown.

Results are reported in Table 3 and visualised in Fig 7. A difference in FSN is seen for the choice of thresholding method, the sample size of the individual studies and the number of peaks. The results for voxel-and cluster-level Family-Wise Error Rate are comparable to one another, especially in the most ecologically valid scenario with a random number of peaks per study. In the scenario of 1 peak per study and voxel-level Family-Wise Error Rate correction on average 3% of the studies in the meta-analysis stem from the original meta-analysis, the rest are noise studies. However, in the other two cases this is on average between 6% and 9%. In every condition, less than 1/10 studies contribute to the activated cluster. A possible explanation for the robustness of the results is that the reported peaks lie close to each other, which results in a large ALE-value.

**Table 3:** Average (and standard deviation) of the Fail-Safe N for different simulation parameters.

	Number of peaks per study								
	1 peak per study			8 peaks per study			Random number of peaks per study		
Type of thresholding	Average sample size			Average sample size			Average sample size		
	10	20	30	10	20	30	10	20	30
vFWE	99.75 (1.41)	99.86 (1.14)	99.87 (1.18)	38.48 (18.32)	40.18 (12.30)	41.56 (12.45)	30.05 (7.38)	35.74 (7.13)	37.73 (7.39)
cFWE (unc)	75.81 (9.32)	87.54 (7.74)	92.59 (9.02)	34.85 (4.02)	41.69 (6.33)	47.06 (6.38)	30.59 (7.87)	37.15 (9.17)	40.84 (10.35)

A positive effect on robustness can be observed for sample size. For voxel- and cluster-level Family-Wise Error Rate correction the FSN of meta-analyses with bigger sample sizes is larger.

The number of peaks has an important impact on the number of noise studies that can be added and interacts with the selected thresholding method. Significant effects are mostly driven by a small proportion of studies in the extreme case of 1 peak per study. A ceiling effect can be observed because we did not add more than 100 noise studies. Adding 100 noise studies to a meta-analysis of only 3 studies implicates that less than 3% of the studies in the meta-analysis contribute to a significant effect.

We downloaded all papers present in the BrainMap database on 26/10/2017. After listing the reported number of local maxima in every experiment we see that on average for every experiment 8 peaks are reported. In this more realistic scenario, the FSN drops for every condition and the results for voxel- and cluster-level FWE are comparable. The average FSN in the third version, where a random number of peaks sampled from the real distribution of the BrainMap database, does not differ a lot from the average of the second version with 8 peaks per study. There is however a greater variability present in the results, again showing that results depend on specific parameters of the meta-analysis.

## 5. Discussion

In this study, we focus on a specific form of publication bias, the file-drawer problem. Publication bias occurs when the results of published and unpublished studies differ significantly. In the case of the file-drawer problem studies that do not show statistically significant results or show results that do not match the hypothesis of the researcher fail to get published. We proposed an algorithm that implements the principles of the Fail-Safe N (FSN) for ALE meta-analyses of fMRI studies, providing a measure for assessing robustness of a statistically significant cluster.

Through an ALE meta-analysis on finger tapping, we illustrate how the algorithm can be used to obtain the FSN. The ALE analyses resulted in 10 statistically significant clusters. Noise studies are generated that resemble the studies present in the meta-analysis as much as possible. The FSN is computed for every cluster and is shown to diverge strongly. While for most clusters the FSN exceeds the minimum and for 6 studies even the predefined maximum, one cluster only requires less than 11 noise studies to be rendered statistically non-significant. Differences in robustness of the various clusters is shown, previously difficult to ascertain even if volume and extrema value are taken into account.

Simulations show that thresholding method, individual study sample size and number of peaks influence the resulting FSN. The results also show the importance of large sample sizes. fMRI studies tend to employ small sample sizes, mainly due to the high costs of executing a study, but this has several disadvantages. First the power will subsequently drop significantly, making it more difficult to detect an effect. As typically only peak locations are reported this increases the chance of consistent small effects remaining undetected. Second, small studies are prone to employ a more lenient threshold to compensate for low power. This leads to an increase in false positives and more noise in the meta-analysis. The FSN assesses the impact of a specific form of publication bias, the file drawer problem, but the impact of selective reporting (less than 1% of data obtained in an fMRI study is reported) cannot be ignored in this respect. Researchers should therefore verify whether an activated cluster is mainly driven by large or small studies and interpret results accordingly.

We also note that the number of peaks has a significant influence on the FSN. Depending on the software used for the fMRI data-analysis and the thresholding method, some papers consistently report more peaks than others, and therefore have a bigger influence on the results of the meta-analysis. While some studies report 1 activated peak for a relatively large cluster, other studies might report 3 peaks inside a relatively small cluster. The ALE algorithm compensates this by taking the maximum of overlapping kernels within the same study (in the MA-map), but it is impossible to completely neutralize this reporting bias.The algorithm to generate noise studies generates peaks at random locations throughout the brain but does not specifically allow the generation of multiple peaks per cluster. While a peak that lies very close to another peak has limited influence on the meta-analysis, as is also illustrated in Eickhoff et al. [28], peaks that are spatially close but with non-overlapping kernels will influence the null distribution of ALE-values used in the thresholding phase because the number of contributing peaks and voxels will change (with more peaks, the more conservative the threshold becomes). The distance between peaks however does not influence the null distribution. This implies that a user can adapt the number of peaks to resemble scenarios where multiple peaks per cluster are reported but it is not necessary to manipulate the distance between generated peaks. When kernels around peaks partly overlap, thresholds will become slightly less conservative as compared to fully separated peaks but this has limited consequences on final results.

The FSN can likewise be computed on results of other coordinate-based meta-analysis techniques. A researcher merely needs to generate noise studies through the tool and put the coordinates in a format adapted to the respective toolbox. These noise studies can then be used to determine the FSN. In principle meta-analysis coordinates, and therefore also coordinates of noise studies, can easily be transformed to a format fit for MKDA or seed-based d mapping. However, since each technique for coordinate-based meta-analyses entails a different approach to combining results of fMRI studies, it might be sensible to conceptualise the FSN, and in particular the contra-evidence, in a different way. For example, while studies with no activation peaks do not alter ALE results, they might have an impact in the other approaches.

One might question the existence of the file drawer problem in the field of fMRI. fMRI studies may suffer from low statistical power to detect a true effect (underpowered studies). To compensate, thresholds that are lower than conventional (e.g. uncorrected for multiple testing) may be used to obtain positive results. While such practices contribute to the abundance of false positive findings in literature, they do not preclude the existence of publication bias. Both types of biases are symptoms of the focus on positive findings. It is important to realize that even when statistical power is high (either due to lenient thresholding or large sample sizes), even true effects will remain undetected in some studies. Furthermore, in the context of fMRI, a study failing to get published might have other causes, such as obtaining statistically significant results that are not in line with a hypothesis (e.g. when activity is found in other regions contradicting existing models). While the extent of the file drawer problem in fMRI is currently largely unknown, recent work in [37] aims to answer this question and provides means for investigating its existence.

While the FSN does not directly quantify to which degree the meta-analysis may suffer from the file-drawer problem, it provides the possibility to assess the robustness of the meta-analysis against this form of publication bias and can be used for CBMA methods that take peak location into account. Further, it would be of great interest to directly assess the amount of publication bias and to correct for it. In classical meta-analysis techniques (based on summarizing effect sizes), this is for instance possible with the trim and fill method [41], that looks at the asymmetry present in a funnel plot and tries to fill the gap. The method of Vevea and Woods [42] corrects for publication bias by adjusting weights assigned to different studies based on study characteristics that could influence likeliness of publication, such as statistical significance. While such methods could be translated to neuroimaging studies as shown in [27] for a single outcome such as brain volume or correlation between regions or to corrections at voxel-level when effect sizes are used as in seed-based d-mapping [33], extending this to settings where only peak locations instead of effect sizes are used is not straightforward.

In this paper, we focussed on the file-drawer problem, though other forms of publication bias exist as well. The FSN assesses the impact of a specific form of publication bias, but the impact of selective reporting (less than 1% of data obtained in an fMRI study is reported) cannot be ignored.

## 6. Conclusion

Meta-analyses are frequently used to combine fMRI data over studies and labs to further build knowledge on the functioning of the brain. Interpretation of final results should therefore also entail assessing how robust results remain in the presence of for example publication bias where only a subsect of selective studies is part of the analysis. We introduce an adaptation of the Fail Safe N (FSN) in the meta-analysis of fMRI studies that greatly aids researchers in assessing robustness of their results. This is the first algorithm that allows assessing cluster robustness to noise and the cluster’s sensitivity to publication bias. For every cluster resulting from the fMRI meta-analysis the FSN can be computed, showing the number of noise studies that can be added before the cluster is no longer statistically significant. We provide a tool that generates noise studies that are based on the number of foci and sample sizes of the studies that are originally entered into the meta-analysis. This tool is accompanied by a step-by-step guide on how to compute the FSN, facilitating its computation. The process is further illustrated in a comprehensive example provided in this manuscript.

We additionally show the influence of different study parameters on the FSN through extensive simulations. The simulations confirm that robustness increases with sample size and that number of foci has a strong influence on the FSN. By showing the influence of individual study parameters such as number of foci and sample size on the FSN, we mark the importance of generating noise studies that are similar to the original studies. In conclusion, the adaptation of the FSN for fMRI meta-analysis provides a measure for robustness of resulting clusters and their sensitivity to publication bias which provides an added value to the interpretation of brain regions selected as statistically significant by the ALE algorithm accordingly.

## Appendix

Illustration of the classic Fail-Safe N.

Assume *k* independent studies where for each individual study *i*, a *Z*_*i*_-value and one-tailed p-value *p*_*i*_ is used to test the null hypothesis *H*_*0*_: *θ*_*i*_ = 0, with *θ* representing an effect size. The original calculation of the FSN is based on computing an overall p by adding Z’s [43]:

$${\text{Z}}_{\text{S}}=\frac{\sum_{\text{i}=1}^{\text{k}}{\text{z}}_{\text{i}}}{\sqrt{\text{k}}}$$

Based on this formula, the number of studies N with Z = 0 that are necessary for Z_S_ to become smaller than the Z-value cutoff Z_α_ can be attained.

$$\frac{\sum_{\text{i}=1}^{\text{k}}{\text{z}}_{\text{i}}}{\sqrt{\text{k}+\text{N}}}<{\text{Z}}_{\alpha }$$

This eventually leads to the formula for the FSN

$$\text{N}>\text{k}{\left[\frac{{\text{Z}}_{\text{s}}}{{\text{Z}}_{\alpha }}\right]}^{2}-\text{k}$$

We illustrate the FSN with an example from the book “Publication Bias in Meta-Analysis” [20]. The analysis is based on a dataset from Raudenbush on teacher expectancy, a paradigm where the influence of teacher expectancy on intelligence test performance is measured [44]. The dataset consists of 19 randomized studies, with one-tailed probability values ranging from 0.0158 to 0.9255. The Stouffer sum of ${\text{Z}}_{S}=10.46/\sqrt{19}$ [45]. If we use this information to compute the Fail-Safe N we get:

$$\text{k}{\left[\frac{{\text{Z}}_{\text{s}}}{{\text{Z}}_{\alpha }}\right]}^{2}-\text{k}=19{\left[\frac{2.44}{1.645}\right]}^{2}-19=22.80$$

This means that 23 studies with a z-value of 0 need to be added to render the previously statistically significant results non-significant. This is less than the proposed 105 and caution is advised when interpreting the results about the teacher expectancy effect. Imagine if in total 42 studies were executed but 23 of these studies remain in the file-drawer because they showed no statistical significant effect. Adding these studies to the meta-analysis would reduce the sum of Z_s_ to 0. In another example from the original paper of Rosenthal on interpersonal self-fulfilling prophecies the FSN is N = 3263 where k = 94 [20]. We can be more confident about these results as it seems highly unlikely that 3263 studies remain unpublished.

In classic meta-analysis the FSN is calculated by computing the average z-value or effect size and deducting the number of studies that can be added before this summary statistic is below a certain threshold. In the meta-analysis of fMRI studies however we want to compute the FSN for a cluster where there is statistically significant spatial convergence of foci over studies. Combining these would be challenging. Furthermore typically only the location (and sometimes the height) of voxels that exceed a statistical threshold are reported, which makes computing a summary value even more challenging. The ALE algorithm solves this by computing for each voxel a union of probabilities (peak or not) instead of a sum or average. Therefore the classic implementation of the FSN cannot be applied here.
